# Cloud Computing Enabled Big Multi-Omics Data Analytics

**DOI:** 10.1177/11779322211035921

**Published:** 2021-07-28

**Authors:** Saraswati Koppad, Annappa B, Georgios V Gkoutos, Animesh Acharjee

**Affiliations:** 1 Department of Computer Science and Engineering, National Institute of Technology Karnataka, Surathkal, India; 2 Institute of Cancer and Genomic Sciences and Centre for Computational Biology, College of Medical and Dental Sciences, University of Birmingham, Birmingham, UK; 3 Institute of Translational Medicine, University Hospitals Birmingham NHS Foundation Trust, Birmingham, UK; 4 NIHR Surgical Reconstruction and Microbiology Research Centre, University Hospitals Birmingham, Birmingham, UK; 5 MRC Health Data Research UK (HDR UK), London, UK; 6 NIHR Experimental Cancer Medicine Centre, Birmingham, UK; 7 NIHR Biomedical Research Centre, University Hospitals Birmingham, Birmingham, UK

**Keywords:** Big data, cloud computing, multi-omics data, data analytics, data integration

## Abstract

High-throughput experiments enable researchers to explore complex multifactorial diseases through large-scale analysis of omics data. Challenges for such high-dimensional data sets include storage, analyses, and sharing. Recent innovations in computational technologies and approaches, especially in cloud computing, offer a promising, low-cost, and highly flexible solution in the bioinformatics domain. Cloud computing is rapidly proving increasingly useful in molecular modeling, omics data analytics (eg, RNA sequencing, metabolomics, or proteomics data sets), and for the integration, analysis, and interpretation of phenotypic data. We review the adoption of advanced cloud-based and big data technologies for processing and analyzing omics data and provide insights into state-of-the-art cloud bioinformatics applications.

## Introduction

**Figure 1.**
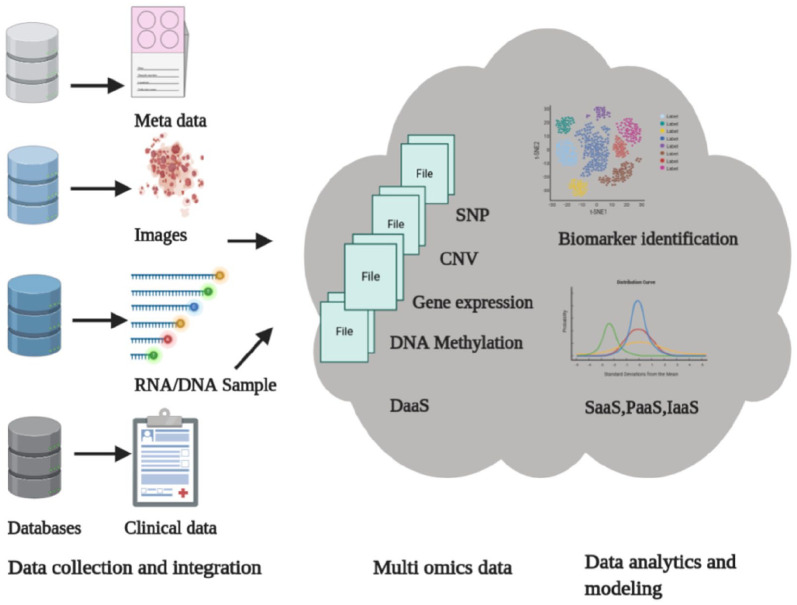
An overview of typical bioinformatics omics analysis framework using cloud computing and big data technologies. CNV indicates copy number variation; DaaS, Data as a Service; IaaS, Infrastructure as a Service; PaaS, Platform as a Service; SaaS, Software as a Service; SNP, single nucleotide polymorphism.

To mitigate data storage and analytical challenges surfaced by the development of omics technologies, over the recent years, numerous novel big data innovations and scalable cloud-based solutions have been proposed and developed. Advanced big data analytics frameworks accelerate the storage and analysis of big omics data by facilitating the provision of scalable analytic infrastructures, such as the Hadoop Distributed File System (HDFS) for storage and the Spark Machine Learning libraries (MLlib) for analysis.^1^ So as to cater advanced bio-data analytics, big data and cloud computing technologies need to be tightly integrated and applied in a uniform fashion. Cloud computing has been demonstrated to be reliably scalable for the analysis of genomic data over single machines, as well as clusters and public cloud infrastructures. The limitations of current data workflows, geared toward high-throughput experiments analytics (called multi-omics data), include security, confidentiality, and limited cloud management technologies. By using multi-omics data available on the cloud, users are able to apply advanced pipelines or workflows, which facilitate their transformation and analysis, reduce the upload and download time while taking advantage of cost-effective computing resources. For example, the Cancer Genome Atlas (TCGA)^2^ project, one of the largest and most complete cancer genomics data sets available, is now making its data available, via an Application Programming Interface (API), on a number of public and private cloud repositories. These efforts provide viable replacements for redundant and costly local infrastructure settings and enable a secure, effective, and reproducible analysis of shared data sets and results. Scalable, cloud-based platforms, such as the National Cancer Institute (NCI) Cloud Pilots program FireCloud, can then be developed that diminish the need for ad hoc, in-house high-performance computing architectures and expensive data transfer.^3,4^ Figure 1 illustrates the use of big data and cloud computing technologies within bioinformatics pipelines, including data collection, data integration, data analysis, and modeling.

**Figure 2.**
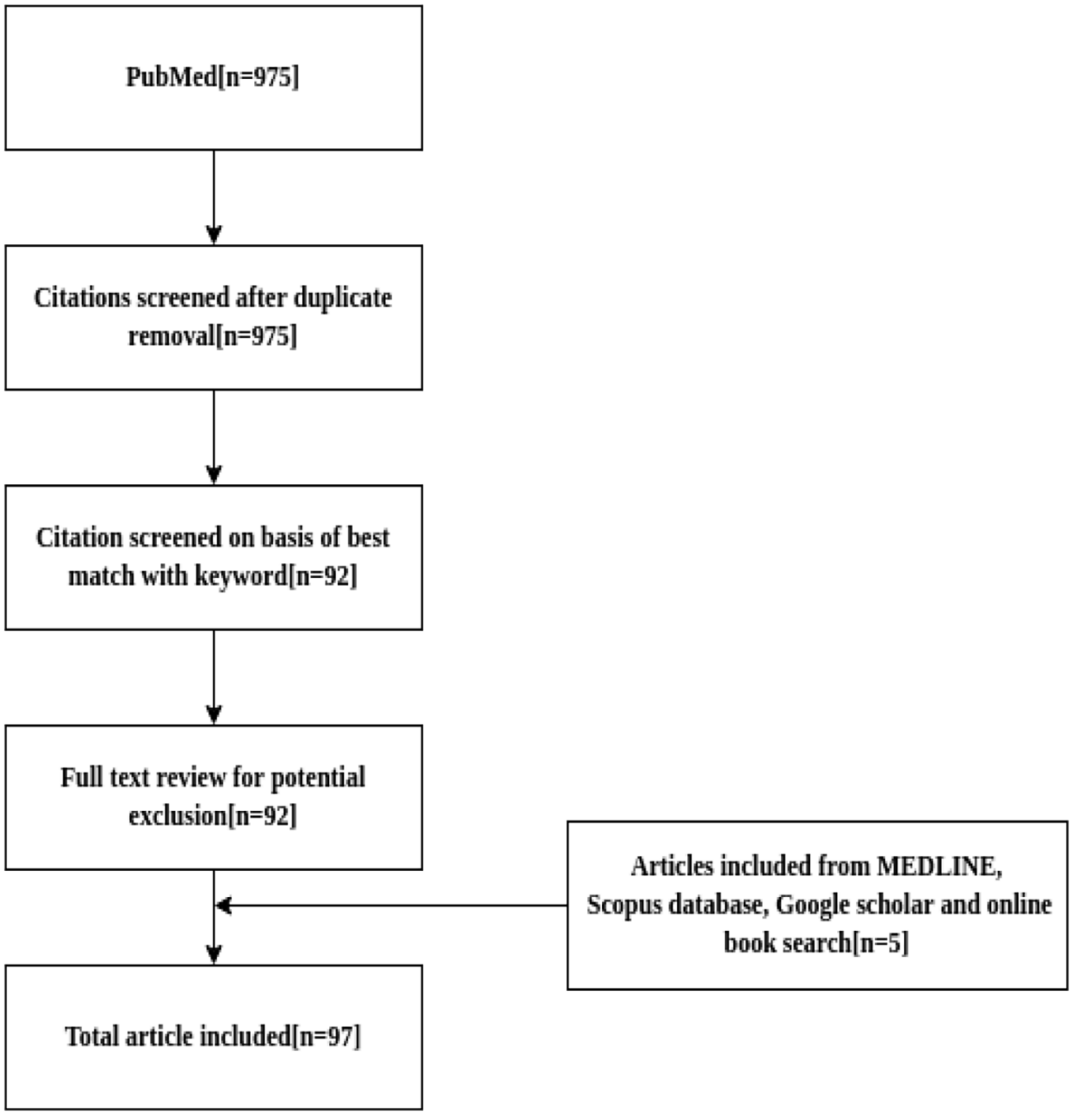
Adopted literature search workflow where “n” indicates the number of articles considered in each of the box resulting in the inclusion of a total number of 97 articles.

Our literature review was carried out across 5 stages (Figure 2), namely, (1) identification and retrieval of relevant publications, listed within the MEDLINE, Google Scholar and Scopus databases, as well as online book search such as Google Books and BookFinder, based on set of specific terms, namely, cloud computing OR bioinformatics OR molecular medicine OR genomics OR multi-omics OR integration OR big data OR cloud computing tools OR big data tools; (2) primary relevance screening (determination of an article meets the inclusion criteria) by selecting the “best matches” option from PubMed based on publication date; (3) review of the relevant papers; (4) summarizing their content; and (5) manual reference screening, to exclude redundant content. Five papers were excluded from our review due to identical title redundancy.

Within this review, we considered the concepts of multi-omics data integration, storage, and analysis frameworks within the context of publications related to the adaption of cloud computing and big data analytics within the molecular medicine and genomics research areas (Table 1).

**Table 1.** Literature search process using specific keyword.

Search database used	Keyword used	No. of documents found	No. of documents included
PubMed	Cloud computing in bioinformatics	460	33
	Multi-omics data integration	329	24
	Big data analytics in bioinformatics	138	20
	Big multi-omics data analysis	38	07
	Cloud computing with multi-omics data	5	03
	Big data analytics tools in multi-omics data analysis	02	02
	Cloud computing tools in multi-omics data analysis	02	02
	Cloud computing and big data tools in multi-omics data analysis	01	01
Included articles available from the MEDLINE, Scopus Google scholar databases as well as online book search such as Google Books and BookFinder	Additional references identified by other databases	20	5

Our review is organized around 2 primary objectives.

1. To review the main bioinformatics concepts, standards, terminologies, and paradigms related to biomedical big data integration, analysis, storage, and cloud computing.
2. To provide an account of the main characteristics, advantages, disadvantages, and differences across multiple cloud-based tools.

## Cloud Computing in Bioinformatics

### Biomedical and multi-omics data: introduction

The exponential growth of biomedical data sets over the recent years has resulted in the identification of a wealth of molecular signatures vital for the realization of the personalized diagnosis and treatment era.^5^ Bioinformatics researchers typically use multiple data from different platforms, such as genomics, proteomics, transcriptome, epigenomics, metabolomics, and imaging, in conjunction with clinical data derived across different modalities, from structured to semi-structured and unstructured. As a result, large-scale and complex data sets are increasingly being considered resulting in several challenges. For example, existing next-generation sequencers produce over 100 GB of raw sequence reads per genome. Together with various clinical and phenotypic features, these data can greatly improve our knowledge of complex diseases but present storage and bioinformatic analysis challenges. Appropriate storage infrastructures capable of hosting such biomedical data can then be exploited to cater applications that exploit their features so as to formulate novel hypotheses related to disease prevention and treatment. Undeniably, nevertheless, big biomedical data tools and technologies currently have a limited translational impact in clinical care. Biomedical big data offer the tantalizing possibility of aiding the identification of novel and key molecules and disentangling their biological and physiological roles and functions. Moreover, their effective use can potentially aid clinical decisions, effective disease treatment, and so on, ultimately improving health care.

Multi-omics data sets derived by the 4 major omics technologies, namely, genomics, transcriptomics, proteomics, and metabolomics, ultimately represent in-depth characterizations of interactions between genes, proteins, and metabolites. There is a need for integrating different omics data for a systematic, in-depth characterization and understanding of the biological processes, eg, those related to adverse outcomes and typical multi-omics studies pertain to the integration of different omics types in an effort to gain a better understanding of the overall complex underlying biological mechanisms.^6-11^ Various platforms are available to profile whole genomes using many samples, enabling a better understanding of complex diseases, like cancer, and complex phenotypic traits. Some of the molecular experimental omics technologies are based on high-throughput mass spectrometry, microarray, RNA sequencing, and DNA sequencing. The analysis of the resulting large-scale data necessitates advanced bioinformatics software or pipelines. Typically, the analysis of omics data involves the imputation of raw data, noise elimination, and identification of relevant features. Other examples of computational pipelines revolve around comparing DNA sequence fragments, or an entire chromosome, with a reference genome to identify variations. Table 2 provides some examples of the various data types that are used in multi-omics profiling.

**Table 2.** The platforms available to provide global multi-omics profiling information in the cloud framework.

Omics type	Platform	Size of each data type can accommodate (approximately)	Cost of analyzing omics data in cloud (approximately)
Genome^12,13^	DNA sequencing	>100 GB (BAM and VCF files)	Per GB storage and transfer rate ranges from $40/test to $66/test
	DNA methylation (array based)		
	SNP based		
Transcriptome^14^ (Total RNA)	RNA-seq	>2000 samples	US$1.30 per sample
	microRNA sequencing (miRNA)		
Proteome^15^	Protein mass spectrometry	Standard mix proteomic data set	Cost of database search using virtual system over cloud is >US$1
	RPPA		
Metabolite^16^	Metabolite mass spectrometry	~1 GB	Resources to process ~1GB of 13 C-MFA data are $11
Microbiome^17,18^	Ribosome RNA (rRNA) gene sequencing and shotgun MGS	>90 GB (FASTQ data)	Library preparation ~$400 sequencing costs ~$8 per GB

Abbreviations: BAM, Binary Alignment/Map; MFA, Metabolic Flux Analysis; MGS, metagenomics sequencing; RPPA, Reverse Phase Protein Array; rRNA, ribosome RNA; SNP, single nucleotide polymorphism.

### Biomedical and multi-omics data sources

Implementing a large-scale data environment to analyze large-scale genomics data in health care necessitates the effective combination and application of various technologies, such as artificial intelligence,^19^ parallel processing techniques, such as Hadoop MapReduce, and data mining tools. Several large data applications, such as the Apache Hadoop software library, are used in biomedical research to overcome scalability, accuracy, and computational complexity issues.^20^ Cloud computing helps data scientists by providing access to computing frameworks, such as the Microsoft Windows Azure platform (https://azure.microsoft.com/en-in/), and to cloud services that can be used to develop particular tools or applications. Adopting and efficiently implementing public cloud repositories to store genomic and patient health information involves critical privacy and security issues. The majority of such public cloud repositories are the result of community-based efforts typically suffering from data curation quality, privacy, and security issues and present complexity and sustainability challenges.

Typically, multi-omics frameworks rely on traditional statistical techniques for data retrieval, integration, and analysis. Such traditional approaches suffer from scalability, time, computational efficiency, and accuracy limitations.^21^ At present, sequence alignment and mapping of high-throughput sequencing data sets remains time-consuming. The numerous de novo assemblers that have been developed, some of which based on message passing interface (MPI) (eg, Ray,^22^ ABySS,^23^ and SWAP-Assembler^24^), exhibit limited scalability, accuracy, and computational efficiency. In addition, DNA analysis pipelines designed to address scalability, such as Halvade,^25^ are characterized by several limitations, including accuracy, and computational efficiency. Similar limitations are aberrant within the single-cell RNA sequencing domain.

The advantages of parallel computation frameworks include high availability and parallel processing, with data being processed by multiple machines, significantly reducing processing times. By bringing computation to data (data locality), the cost of moving processing units to data resources is removed, and processing times are reduced because all cluster nodes can work in parallel and simultaneously. Large data frameworks, encompassing parallel processing and in-memory processing, achieve higher memory efficiency.^26^ As a result, data scientists commonly use big data analytics tools, such as Hadoop to store data, MapReduce for data analysis, and use tools such as Pig (https://pig.apache.org/) and Hive (https://hive.apache.org/) for data retrieval. Such tools are frequently used in conjunction with several open-source tools, eg, R, Python, and scalable machine learning tools, and commercially available tools, eg, MS SQL, Tableau, and Oracle Rdb.^27^ Table 3 lists some examples of different tools along with their advantages and limitations.

**Table 3.** Summary of big data tools for genotype and other omics analysis.

Application	Tools	Description	Advantages	Limitations
Genomic sequencing analysis	Crossbow^28-30^	A pipeline for whole-genome re-sequencing analysis, combining Bowtie and Soapsnp	Cost-effective, automatic, memory-efficient and ultrafast short-read aligner	Single cluster implementation Postalignment bottleneck due to insufficient thread use during multithreading
Programming model	Dryad^31^	A parallel processing framework with the extension of MapReduce for NGS data analysis. Runs on Hadoop YARN	Easy implementation over large data clusters	Works solemnly on DAG and renders the development of new models challenging
Short-read mapper	DistMap^32^	A scalable, modular, and unified workflow for mapping short reads from NGS data in the distributed Hadoop computing framework.	Rapid parallel processing and accurate analysis using parallel graph algorithms	The 2-step input output transfer requires huge amount of disk space
Proteomic search engine	Hydra^33^	A scalable proteomic search engine for high-rate data generated from mass spectrometry. Runs on the Hadoop MapReduce framework	Use of the Hadoop infrastructure, catering the management of parallel jobs by reducing infrastructure costs	Scalability issues due to increasing search rates with increase in mass spectrometry proteomics
Phylogenetic analysis	GATK^34^	A framework for large-scale next-generation DNA-sequencing analysis using MapReduce	Use of a robust common data management engine. Provision of automatic parallelization with efficient memory and CPU utilization. Applicable to both shared memory and distributed machines	Does not support additional data access patterns
Sequence file management	Hadoop-BAM^35^	A novel scalable distributed processing library uses the Hadoop framework for manipulating aligned next-generation sequencing large-scale data	Use of Picard SAM JDK. API to implement MapReduce to operate on BAM records, Picard API easily supports large-scale distributed analysis	Uses command line, which is not user-friendly and limited in scope; nonexpert Hadoop users face difficulties
Query engine	SeqWare^36^	Query engine used to load and query variants with a rich annotation standard, including coverage and functional consequences. Built with NoSQL HBase database.	Helps build automated workflows and processes for large-scale NGS analysis. SeqWare tracks analytical events by linking to samples and studies	Does not work well if you want to analyze small number of NGS samples. SeqWare does not contain pre-built workflows to analyze NGS data sets
Phylogenetic analysis	MrsRF^37^	A scalable multicore algorithm computing the Robinson-Foulds (RF) distance matrix between a large numbers of (t) trees using the MapReduce for multi-core phylogenetic applications	The MapReduce framework reduces output size of all-pairs RF distance (t × RF matrix), therefore advantageous in computations involving phylogenetic tree	MrsRF does not incorporate communication cost
Phylogenetic analysis	Nephele^38^	A tool suite that uses a composition vector algorithm for sequence comparison and affinity propagation clustering for grouping sequences into genotypes. Provision of an advanced computing infrastructure for understanding role of microbiota in human health by Amplicon-based and whole metagenomic sequencing analysis	Cost-effective. All jobs in analysis are reproducible. Tracks input files, VM images used in data analysis	Limited granular control of parameters and flexibility in output generation
GPU-based software	GPU-BLAST^39^	An 4 times faster version of NCBI-BLAST	Capable of using both GPU and multiple-core CPU for parallel execution of comparisons of short and long sequences	High power consumption. Load balancing required gaining higher performance with large clusters
GPU-based software	SOAP3^40^	The first parallel short-read alignment tool used to improve speed and deployed on multi-processors in GPU	2 to 10 times faster than widely adopted sequencing tools, achieves highest sensitivity and low false discovery rates on different length sequence reads	Limited to INDELs, and small gaps identification, alignment reads up to 4 mismatches
Hadoop-based framework	Biodoop^41^	A Hadoop-based framework for the generation of large-scale virtual clusters for sequence alignment	Computational efficiency, scalability, and maintenance	Start-up overhead, improvement in post-processing of BLAST results and parallelizing computation of *P* value
Large-scale sequencing	BioPig^42^	A novel sequence data analysis framework for bioinformatics applications using MapReduce and pig Latin	Automated scalability with exponentially growing sequence data	Slow start up of MapReduce jobs
Feature-rich sequence processing	SeqPig^43^	Scalable and simple scripting for parallelizing large-scale sequencing tasks on distributed Hadoop that uses Apache Pig scripting language	Automatic scripting for parallelized data processing	Implementing interactive jobs are impossible due to MapReduce
Workflow	Nextflow^44^	Open-source workflow framework used for scalable and integrative data-intensive bioinformatics computational pipelines	Software containers are used to enable consistency and reproducibility. Built-in support for HPC environments, singularity, and docker support. Portable, fast prototyping, scalable, and stream oriented	Does not support the CWL specification, module, workflow compositions There is no implementation of a graphical user interface to interact with the pipeline Does not spawn the executions of pipeline tasks through a distributed cluster such as Apache spark
Workflow	Snakemake^45,46^	Designed for reproducible and scalable data analyses	Provides an execution environment that scales to server, cluster, grid, and cloud environments without modifying the workflow definition	Automatic translation of any CWL workflow definition into a Snakemake workflow not yet implemented
Parallel RNA-seq processing	Falco^47^	Cloud-based framework to enable parallelization, RNA-seq alignment/feature quantification, and quality control using big data technologies of Apache Hadoop and Apache Spark	Usage of spot computing resources for analysis provides a ~65% reduction in the cost of analyzing data	Large files splitting speed

Abbreviations: API, Application Programming Interface; BAM, Binary Alignment/Map; CPU, Central Processing Units; CWL, Common workflow language; DAG, Directed Acyclic Graph; GATK, Genome Analysis Toolkit; GPU, Graphics Processing Unit; GPU-BLAST, General-purpose graphics processing unit Basic Local Alignment Search Tool; HPC, high-performance computing; MrsRF, MapReduce Speeds up Robinson-Foulds; NCBI-BLAST, National Center for Biotechnology Information–Basic Local Alignment Search Tool; NGS, next-generation sequencing; RF, Robinson-Foulds; SOAP3, Short Oligonucleotide Alignment Program 3; VM, virtual machine.

There are numerous publicly available data sources that cater the storage, indexing, and provision of omics data sets, offering a variety of analysis and visualization tools. For example, in 2005, the Cancer Genome Atlas (TCGA) and 2008 International Cancer Genome Consortium (ICGC) projects released comprehensive cancer genomics profiles using new analysis technologies and made them freely available within a number of repositories (see Supplementary Table S1 for more recent examples of omics data sources).

### Cloud computing terminologies

**Figure 3.**
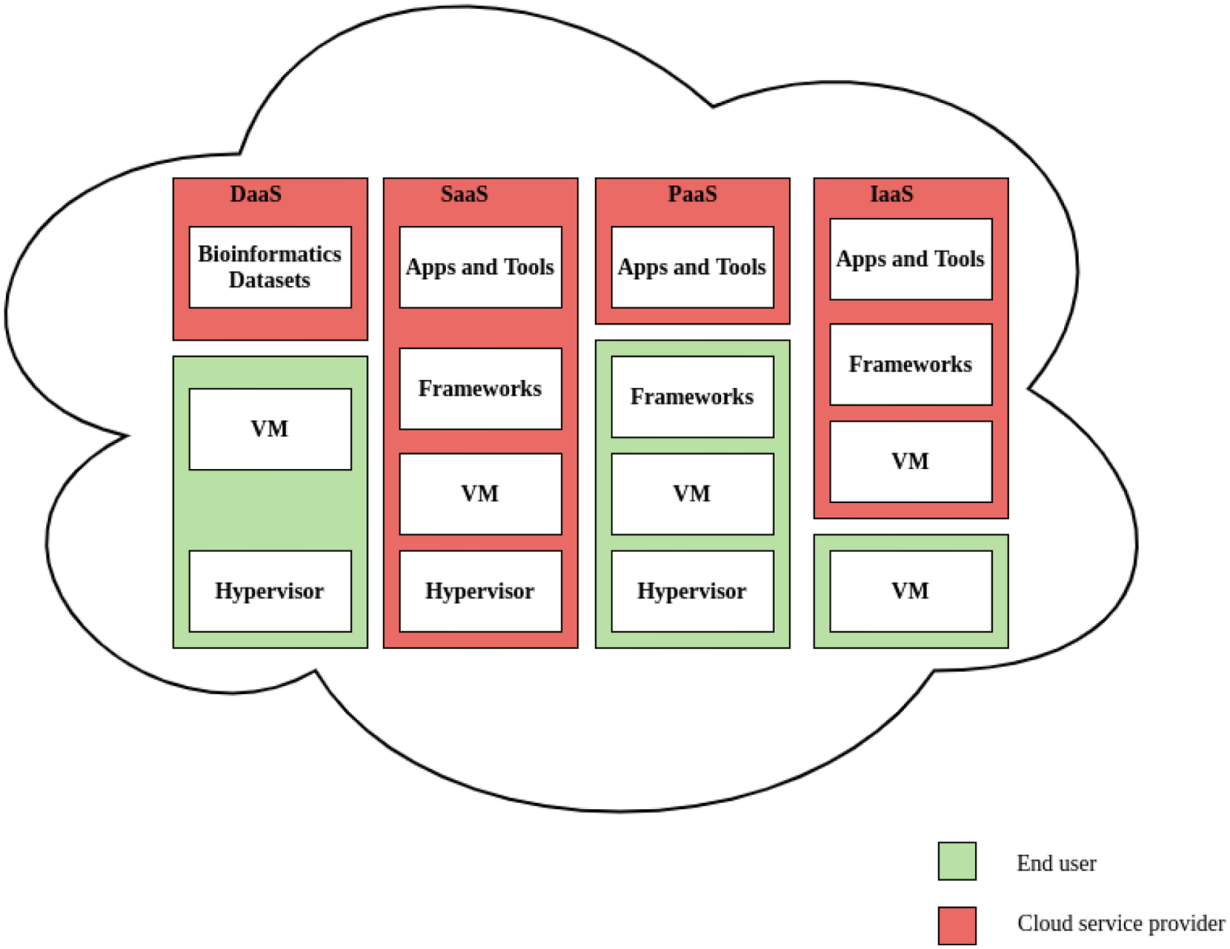
Bioinformatics cloud computing models DaaS, SaaS, PaaS, and IaaS and their distribution services. DaaS provides bioinformatics data sets as services in dynamic virtual space over a network (cloud), end-user can use VM and hypervisors for cost-effective storage and large-scale data analysis. Apps and tools represent cloud-based data exploration, visualization, and analysis tools used in different layers of bioinformatics analysis pipelines (SaaS). Frameworks represent the collection of deployment and management tools required for different bioinformatics tasks (PaaS). IaaS includes computing infrastructure in terms of virtual servers and bioinformatics applications for storage and analysis. DaaS indicates Data as a Service; IaaS, Infrastructure as a Service; PaaS, Platform as a Service; SaaS, Software as a Service; VM, virtual machine.

The National Institute of Standards and Technology (NIST) characterizes cloud computing as a model for enabling ubiquitous, convenient, on-demand network access to a shared pool of configurable computing resources (eg, networks, servers, storage, applications, and services) that can be rapidly provisioned and released with minimal management, effort, or service provider interaction.^48^ The cloud computing model is composed of 5 key features: (1) resource pooling, (2) on-demand service, (3) broad network access, (4) rapid elasticity, and (5) measured services. In addition, cloud computing also comprised 3 service models, namely, Software as a Service (SaaS), Platform as a Service (PaaS), and Infrastructure as a Service (IaaS), plus 4 deployment models: private, public, hybrid, and community clouds. The main essential characteristics of Cloud computing are scalability, redundancy, reliability of hardware, cost-effectiveness, robustness, flexibility for data, and applications.^48^ Within the bioinformatics domain, cloud-based services adopt the above categorization and are typically grouped as Data as a Service (DaaS), SaaS, PaaS, and IaaS^49^ (Figure 3).

#### Data as a Service

Data as a Service is a cloud strategy used to provide and distribute on-demand access to biological data over a network for analysis and knowledge discovery. The objective of DaaS is to overcome data access limitations the current state-of-the-art approaches face by enabling the user to store and access data from any location for sharing and processing. Stephens et al^50^ compared big genomics data with other sources of big data generation, such as business, social network, and the Internet of things and found that genomics data will become much more extensive concerning creation, storing, processing, analyzing, and transmitting by 2025. Biological data acquisition is distributed and heterogeneous, which reaches 1 zetta-bases. Biological data distribution extends from a few base comparisons, or many small transfers of gene sequences (10 MB/s), to fewer large multiples of terabyte (10 TB/s) bulk transfer/downloads from central repositories. Due to its ability to overcome access limitations, DaaS is the most important biological study service that provision big data. For example, Amazon Web Service (AWS) is a cloud-based application that provides data as a service, which gives dynamic access to public data sets to users on demand. AWS includes publicly available data sets from multiple sources, including large biological resources, such as Ensembl^51^ and GenBank.^52^

#### Software as a Service

Software as a Service is a cloud computing facility where users can dynamically access applications online. As bioinformatics studies typically encompass multiple data types, it is important to access up-to-date applications on demand to process them through user-friendly interfaces such as Microsoft 365 (https://www.ncitech.co.uk/business/cloud-computing/microsoft-office-365). Some examples for cloud-based SaaS solutions for genome resequencing include rainbow, short-read aligner CloudBurst, variant annotation VAT, and RNA-seq Myrna.^53^ These tools have several advantages and limitations (Table 4).

**Table 4.** Summary of cloud-based bioinformatics tools for genotype and other omics analysis.

Tools	Description	Advantages	Limitations
DaaS			
AWS Public Data Sets^70^	Access to controlled repositories and public data sets such as TCGA, dbGaP	Efficient data storage, access, and computation. Scalable solutions for genomic analysis acceleration	Limited security features
SaaS			
Myrna^71-73^	A tool to calculate differences of gene expression data from RNA-seq data sets. Can be combined with elastic MapReduce on local Hadoop or single computer	Rapidly tests multiple models for publicly available RNA-seq data sets. Bowtie is used for short-read alignment	Does not attempt to align reads across junctions, assemble isoforms
CloudBurst^74^	Parallel read genome mapping algorithm with MapReduce	Facilitates scalability, automatic monitoring, redundancy and high-performance distributed file access. Faster and more efficient with short-read mapping	Lower accuracy as mismatch mapping not implemented. Proved to be slow with respect to processing time. Designed to work with small reads so unable to manage long reads
BlastReduce^74^	An optimized short-read mapping algorithm for efficiently identifying alignments with small differences. Hadoop MapReduce implementation for parallelizing execution over multiple compute nodes	Identification of sequences for penalized genomics, SNP discovery and genotyping	Handles short-read data. Hadoop has limitations of high I/O time during different iterations. Need for robust hardware and software tools for better time optimization
Rainbow^75^	Analysis of genomic sequencing data from a large number of subjects (>500) in the Amazon cloud	Provides load balancing and automation of WGS data analyses. Able to handle both BAM and FASTQ input files. Able to scale up and down reliably, enabling shorter analysis time regardless of sample size	Not cost-effective as it uses Amazon cloud service (~US$120 to analyze each sample). Difficult to handle network congestion and traffic during large data transmission
eCEO			
FX			
RSD			
VAT^76^	Provides novel visualization of functional annotation variants across different genomes at the transcript level; obtains statistical summaries across genes and individuals	Able to annotate MNPs and offers unlimited storage capacity	Lack of support for determining variant effects using ensemble gene models. Variant location description but lack of biological interpretation
SEAL^77^	A tool suite producing short-read pair mappings that are consistent with BWA mappings	Uses Picard Mark Duplicates criteria for removing short-read pairs duplicates	Supports for short-read mapping only
CloudBrush^78^	A de novo distributed genome assembler based on string graphs with novel edge-adjustment algorithm and MapReduce	Edge-adjustment algorithm helps in finding structural defects (sequencing error) and regulates the edge of the string graph	Only supports batch-based data. Small data sets were used for evaluations. Due to Hadoop’s disk-based computing (ie, massive disk I/O), its performance degrades when dealing with extremely large data sets
Cloudgene^79^	A MapReduce-based GUI framework for large-scale data processing on a cluster (public cloud) and workflow reproducibility over private clouds	Cloudgene can be run on private clusters, allowing for the protection of sensitive data sets, reducing data transfer times	During job concatenation, it is not possible to execute specified pipeline steps automatically. The cluster architecture is static and cannot be altered during operation
Cumulus^80^	Cumulus is a cloud-based framework for analyzing single-cell and single-nucleus RNA-seq data	Scalable, cost-effective, able to process multiple data types	Only Dockers and WDL are used on the Terra platform and Google Cloud Platform
PaaS			
Eoulsan package^81^	High-throughput sequencing data analysis tool on cloud computing services for batch analysis	Automatic and unique analysis solution for several samples	Specifically targets RNA-seq data analysis. Not emphasizing on graphical job execution on public and private clusters
Galaxy Cloud^82-84^	A cloud-based framework for genomics research ensuring the reproducibility of large-scale analyses	Provides free and open solutions for reproducibility, dissemination, and generalized reuse problems by capturing execution information to understand complex computational analysis Provides integrated tools for a variety of biomedical studies	Difficult to adopt specific analysis tools. Moving large amount of data reliably and efficiently is challenging
SparkSeq^85^	Scalable and fast tool for interactive next-generation data querying with nucleotide precision using Apache Spark and MapReduce	Interactive, parallel, in-memory ad hoc data exploration option. Users can speed up and optimize larger data analysis by running and tuning parameters several times (when multiple samples are present)	Lack of alignment options and of batch NGS-data processing. Lack of CRAM and ADAM file formats support
IaaS			
CloVR^86^	Single portable VM sequence analysis application provides an automated sequence analysis pipeline	Remote cloud computing services option	Relies on BLAST for sequence matching and taxonomy assignment
Cloud BioLinux^87^	Publicly available cloud framework for developers to create and share customized virtual machines for high-performance bioinformatics applications	Uses VMs whole system snapshot exchange feature Computing resources, such as OS, databases, and other software tools, are encapsulated into a single image for later use	Uses a publicly available cloud framework
BPDC^88^	Open-source cloud platform based on OpenStack contains petabytes of genomics and phenotypic data, tools, and computing resources such as virtual machines	Provides a high-performance cluster file system (GlusterFS) allowing users to access large genomics data sets to their working space	Use of unsecured public or external devices for data transfer
Galaxy CloudMan^89^	Cloud resource management system. Provides solutions for configuring compute clusters on Amazon’s EC2 cloud infrastructure to perform bioinformatics analysis for researchers	Use of multiple cloud infrastructures, such as AWS, OpenNebula and Openstack. Allows custom deployment of resources like arbitrarily sized clusters. Provides dynamic scale-up and scale-down resource allocation	Not cost-effective. Requirement to pay for cloud resources used. Lack of default MapReduce support prevents graphical executions
CloudAligner^90^	MapReduce-based tool for genome sequence mapping generated by next-generation sequencing	High-performance gain due to parallel processing and partition of the large reference genome and long reads (used seed-and-extend algorithm)	Lack of stream of reads alignment
CloudBLAST^91^	Parallelization and management of bioinformatics applications using NCBI BLAST on WAN-based clusters, by integrating Hadoop MapReduce and virtualization technologies for distributed computing	Support for customization, integrative, and flexible solutions for variety of problems	Relatively low weight computation on large data sets
Nextstrain^92^	Nextstrain is an open-source project consisting of a database of viral genomes, a bioinformatics pipeline and interactive visualization platform for phylodynamics analysis	Advanced computing environment AWS Batch, which allows users to launch and monitor more reproducible Nextstrain build in cloud	Privacy and security issues with visualizing and sharing sensitive or private metadata
BugSeq^93^	A bioinformatics platform delivers rapid, scalable, and automated microbiology sequencing analysis	Accurate and fast metagenomic analysis for nanopore reads	Execution time and high processing requirements for performing full read alignments against all of RefSeq
nf-core^94^	A framework for the development of collaborative analysis pipelines. nf-core genomic pipelines are written in Nextflow. Support for AWS-iGenomes, as well as for container technologies such as Docker and Singularity	Supports execution of pipelines on most computational infrastructures	Simplified interactive command line and graphical user interfaces would be beneficial. Lack of infrastructures to perform automated benchmarking, and more accurate cost estimating tools for cloud computing

Abbreviations: ADAM, Analysis Data Model; AWS, Amazon Web Services; BAM, Binary Alignment/Map; BLAST, Basic Local Alignment Search Tool; BPDC, Bionimbus Protected Data Cloud; BWA, Burrows-Wheeler Alignment; ClovR, Cloud Virtual Resource; DaaS, Data as a Service; eCEO, Cloud-based Epistasis computing; FX, user Friendly gene eXpression; GUI, graphical user interface; IaaS, Infrastructure as a Service; I/O, input-output; MNP, multinucleotide polymorphisms; NCBI, National Center for Biotechnology Information; NGS, next-generation sequencing; OS, operating systems; PaaS, Platform as a Service; RSD, Reciprocal Smallest Distance algorithm; SaaS, Software as a Service; SNP, single nucleotide polymorphism; TCGA, The Cancer Genome Atlas; VAT, Variant Annotation Tool; VM, virtual machines; WDL, workflow description languages; WGS, whole-genome sequencing.

#### Platform as a Service

Platform as a Service is a cloud computing model that provides software tools and hardware to users on demand. It is useful for processing large biological data by dynamically requesting software and hardware environments. The main beneficial characteristic of PaaS is scalability. PaaS improves scalability by providing a working environment over the Internet as and when users demand, allowing users to analyze data sets with many samples by using available resources automatically. PaaS allows batch processing of high-throughput sequencing data. Bioinformatics uses 2 PaaS platform services, Eoulsan and Galaxy, for analysis of large-scale high-throughput sequencing data.

#### Infrastructure as a Service

Infrastructure as a Service is a cloud paradigm that facilitates virtual infrastructure, such as computing, storage, and networking, over the Internet. IaaS now provides databases, messaging queues, and other services on top of the virtualization layer. Examples of IaaS are the Microsoft Azure, the Amazon cloud, the Google computing engine, and the Joyent.^54,55^ To store, compute, and exchange such large data, cloud computing provides PaaS virtual resources over the Internet. There are 2 primary publicly available PaaS virtual machine services for bioinformatics: Cloud Virtual Resource (CloVR) (http://clovr.org) and CloudBioLinux (http://cloudbiolinux.org/). These are portable virtual machines for automated sequence analysis and provide on-demand high-performance environments.

#### Other key emerging cloud technologies and platforms

##### DNAnexus

DNAnexus (DNAnexus, Inc, Mountain View, CA, USA) provides an API-based platform for sharing and managing genomic data and tools to accelerate genomic research benefiting from transparency and reproducibility. DNAnexus has scaled to over 56 000 concurrent computing cores, numerous petabytes of storage, and tens of millions of core hours of analysis using Amazon Web Services. Users can upload raw DNA data straight from sequencing machines to the cloud using both a graphical user interface (GUI) and a command-line tool, avoiding the need for costly, on-premise, processing and storage infrastructures (https://www.dnanexus.com/).

##### DNAstack

DNAstack is a cloud-based platform for storing, managing, and analyzing genomes and other patient data. DNAstack is based on DNA sequencing technology, which has allowed individual genomes to be read, potentially improving diagnoses and treatment. It is part of a Canadian-led program to speed up genomic data exchange worldwide, claiming to be the world’s largest genetic mutation search engine.^56^

##### Terra

Bio platform.

Terra is a cloud-native platform that allows biomedical researchers to interact, access data, and execute analysis tools with security being prominent. It provides a scalable architecture connecting cloud data repositories and enabling researchers to conduct integrative analysis over big data sets in a reproducible manner. Researchers are also provided can with the option of federating various data sets and perform integrative studies (https://app.terra.bio/).

##### Illumina BaseSpace

Illumina has the flexibility to accommodate its users’ need for on-demand research by operating BaseSpace Sequence Hub on AWS. Illumina offers to spin up 2000 instances in just a few hours using AWS, eliminating the need to load a data center with hardware. Workloads can be executed in parallel, without incurring a substantial initial cost. Users can set up runs and assess the quality of instrument runs and computational resources without having to invest through infrastructure. In addition, ease of accessibility to a multitude of genomic analysis software boosts organizational efficiency (https://sapac.illumina.com/products/by-type/informatics-products/basespace-sequence-hub.html).

##### NVIDIA GPU

Genomic data can be analyzed faster, more precisely and at larger scales over GPU-accelerated platforms. Nvidia’s CUDA (Compute Unified Device Architecture) is the most widely used library for developing GPU-based tools in bioinformatics, systems biology, and computational biology. Although CUDA can only be deployed over Nvidia GPUs, there are other options, including Microsoft DirectCompute catering its use in conjunction with Microsoft’s Windows operating system as well as deployment over the platform-independent library OpenCL (which can use AMD/ATI GPUs).^57^

##### Databricks Genomics Platform

The Databricks Genomics Platform offers preconfigured GATK processes, hosted on AWS and Azure, to enable quicker genomic data preparation and processing. Data can be processed 15 times faster when workflows are optimized to operate in parallel and prepackaged genomic analytics, and machine learning frameworks can be used for it interactive evaluation. With autoscaling on AWS and Azure, users can analyze hundreds of thousands of genomes while lowering expenses. Connect processed genetic data to downstream analytics in real time for faster outcomes (https://databricks.com/product/genomics).

##### Cromwell workflow description languages

Cromwell is an AWS Cloud–based workflow execution engine developed by Broad Institute. It renders orchestrating computational operations for genomics analysis much easier, offering considerably more flexibility in scaling genomics research by leveraging cloud computing capabilities rather than competing for limited on-premise resources. Based on the volume and particular resource requirements of the batch jobs submitted, AWS Batch, a fully managed batch computing solution on Amazon Web Services, automatically provisioned the optimal quantity and kind of compute resources (https://aws.amazon.com/government-education/cromwell-on-aws/).

The NCI Cloud Resources FireCloud, Institute for Systems Biology (ISB), and the Seven Bridge Platform are part of the National Cancer Institute’s Cancer Research Data Commons. Data access via a Web-based user interface is available in all 3 NCI cloud resources and provides access to analytic tools and workflows via a programmatic interface and the opportunity to share results with collaborators. Each Cloud Resource constantly adds new features to improve the user experience and provide researchers with new tools. Each Cloud Resource has built its infrastructure, along with a variety of tools for accessing, exploring, and analyzing molecular data. Other data types, like medical imaging and proteomic data, Radiologic and pathology images, are accessible through all 3 NCI Cloud Resources. NCI FireCloud runs on the Google Cloud Platform (GCP). Data uploading, cloning, and creating a new TCGA workspace.^58^ ISB Cancer Genomics Cloud includes processed data in BigQuery and provides cohort comparison and integration services.^59^ Seven Bridges Platform^60^ deployed on AWS provides query system to find exact data and allows researchers collaborative analysis.

### Scope and implementation of cloud computing technologies for multi-omics and for biomedical data analytics

Bioinformatics experimental data continue to increase due to technological growth and reduced cost of the experiments with current resources extending from terabytes to petabytes. The efficient computational analysis of such enormous data sets requires approaches that facilitate their volume reduction. One such an approach lies with the implementation of stringent quality control using post-experimental processing. These implementations, however, require scalable and robust computing solutions, such as the one offered by cloud computing.

Many big data frameworks, eg, Apache Hadoop (https://hadoop.apache.org/), are now integrated with cloud computing to improve system speed, agility, and time to maintain hardware and software resources. The implementation of SaaS, PaaS and IaaS services allows cloud computing approaches to provide dynamic scalable resources that cater different hosting and analysis workloads as virtualization services that operate across different levels of stacks.^61^

#### Virtualization

Virtualization is the generation of an abstract layer of hardware, software, storage, or network resources to ensure maximum utilization of these computing components. Virtualization ensures the reliable use of resources, such as memory, disk storage, and Central Processing Units (CPU), and limits redundancy by the abstraction of individual applications, eg, VMware ThinApp. Virtualization can also be achieved by combining scalable and elastic solutions hosting multiple Virtual Machines (VMs) on a single machine. VMs allow operating systems (OSs) and other applications to run across multiple VMs installed on a single machine. A hypervisor is a virtualization management layer that controls resource allocation. Dynamic (scaling up and scaling down), on-demand resource management helps reduce both the cost and the time required to build and maintain complex computational infrastructure for multi-omics data analysis and storage.^62^ Amazon EC2 provides various VM images, as well as bioinformatics applications, to address data management issues typically encountered by bioinformatics studies.^63^ Other examples of publicly available VMs are the Cloud BioLinux and the CloVR.

Containerization is referred to as lightweight virtualization and allows bioinformatics workflows to accelerate portability and reproducibility and ensure scalability. Container technologies, such as Docker and Singularity, are a component of cloud computing frameworks, with Kubernetes being used to manage container orchestration. Docker is the most extensively used framework allowing users to create, store, and manage Linux-based environments deployed on almost any computer.^64^ Singularity, on the contrary, is a computing framework for providing computational mobility to users and HPC facilities, allowing for the secure acquisition and distribution of software and computing environments. It allows users to execute environments from a range of resources (including Docker) without requiring privileged access. By combining Singularity and Docker, the user may be highly flexible in how, when, and where to use their own and others’ computing environments.^65^

By containerizing applications, the reproducibility and portability are ensured, allowing for sophisticated workflow management solutions, such as Nextflow, to accelerate the generation of portable and scalable pipeline.

#### Time and cost reduction

Cloud computing systems are reliable, scalable, and cost-effective information technology (IT) platforms that are increasingly being adopted for large-scale bioinformatics analysis. They typically use distributed resource management solutions, such as the SunGrid Engine (SGE) and Load Sharing Facility (LSF),^66^ that facilitate a number of tasks, frequently necessitating concurrent execution of processes, such as quality control, alignment, and genomic features extraction. Such features render them ideal to address computational challenges, eg, complexity, and implementation requirements, such as multimode scalability and typical genomic data processing, pipelines face. Moreover, faster heuristic solutions are increasingly becoming available, such as the ones developed for search and sequence alignment. For example, GenBank uses a cloud-based tool, termed BLAST (Basic Local Alignment Search Tool), to query sequences^67^ and reduce the time complexity while decreasing the sensitivity of the resulting alignment.

#### Performance

Cloud computing offers a variety of both CPU and Graphics Processing Unit (GPU) acceleration frameworks for enhanced performance. GPU-based cloud computing is a promising biological data analysis approach as the performance to price (P/P) ratio is more favorable for GPU than for CPU. GPU reduces the cost of hardware and accelerates data processing by using parallel processing over several GPU cores. However, GPU-based cloud applications suffer from the slow data exchange between GPU and CPU due to slow input-output (I/O) operations and the relatively small GPU memory limiting input data storage (Table 4).^68,69^

## Discussion

Recent technological advances have led to the generation of large biomedical data sets of various datatypes generated from different platforms, necessitating interoperable integration frameworks for their analysis. The adoption and use of cloud computing are a promising and viable solution to overcome these challenges due to its virtualization, advanced analytics, storage optimization, and scalability properties. Furthermore, cloud computing facilitates simultaneous multivariable processing, and the development of efficient methods to reduce computational time and memory utilization will be a crucial step-change to systems biology research.

### Bioinformatics big data challenges

Collecting, integrating, and systematically analyzing heterogeneous big data with distinct characteristics are a challenging task that may lead to data mismanagement, raising issues, including privacy, security, and related ethical ones. Big data analytics frameworks are useful in performing a series of tasks in a distributed manner, reducing the hardware workload to overcome scalability challenges, specifically supporting simultaneous high-performance genomics data processing, achieving scalability and reliability, and addressing redundancy issues related to large genomics data processing. Big data frameworks help overcome fault tolerance by replicating data in a distributed manner sidestepping software or hardware failures due to unreliable data replications. Distributed data processing frameworks additional advantages include high availability and distributed data replication for complex systems. Parallel processing, whereby multiple machines simultaneously process data, reduces processing time and presents another advantage of distributed data processing frameworks. Data locality reduces costs and single nodes’ burden. Furthermore, parallel and in-memory processing ensures higher memory efficiency.

### Cloud-based omics challenges

Although cloud computing offers considerable advantages, there are some challenges and limitations. Some of the challenges are related to data privacy and security and can be considered the biggest threat to cloud computing in the health care data analytics domain.^95^ Authentication, authorization, and access control within the cloud’s virtualized network are essential and several data security concerns, including data leakage and loss issues related ones, still need to be addressed.^96^ Some other significant challenges to cloud-based omics relate to infrastructure requirements for systematic analysis and advanced query frameworks of big data sets, which are particularly applicable to large-scale, integrated, heterogeneous bioinformatics data sets that are increasingly becoming available.^97^ Different omics repositories need to be incorporated to provide reliable and practical solutions, and standardized approaches are needed to eliminate their inherent variability. Cloud applications are required to perform tasks alongside distributed data, necessitating interoperability and portability which present a further challenge. Other challenges include the lack of homogeneity, including qualitative and quantitative variables measured at different scales, to characterize a phenotype or trait.

Crucially, while cloud computing is inexpensive, the platform adaption to meet the demands of the users can be costly. In addition, the cost of transferring data to public clouds can be expensive. Cloud computing downtime, which is typically listed alongside system failure, human error, network failures, a lack of resources, and the provision of multicloud environment management and multicloud strategies for building hybrid clouds that combine public and private cloud resources, is critical. Finally, law and regulation compliance, in particular for health-related data sets, is crucial.

Nevertheless, cloud computing allows cost-effective distributed storage and analysis of such large data sets, and it operates on self-deployment models with pay-per-use, on-demand, scalability, and elasticity features. Big data approaches exploit previously ignored data sets, providing valuable insights gained by their ability to exploit data sets that traditional methods cannot interrogate. Cloud computing enables software versatility and speed to streamline such tasks. Big data approaches typically adopt a strategy of splitting large data sets into manageable chunks and distributing them across the various computer systems, helping to parallelize computation over large data sets. Cloud computing allows for storage and analysis on remote physical servers managed and operated by service providers, accessed by the user through the network. So as to deal with performance and scalability for big genomic data, parallel programming models in a distributed environment, such as MapReduce (https://aws.amazon.com/emr/), are increasingly being adopted.

Sequencing technologies continue to decrease costs while the amount of data produced increases. New data processing and storage platforms are becoming more and more essential, and the scaling behavior of these emerging technologies directly impacts biomedical research. It is challenging for data scientists to design and develop practical algorithms in working applications for secure outsourcing of encrypted biomedical data. Moreover, developing standard approaches to enable secure coordination of data integration across multiple sources can be very demanding. The establishment of secure computation frameworks can ensure the efficient analysis of such data sets. Cloud computing practice could solve the big data analysis problem of efficiency in time, memory usage, and storage, albeit it is still at quite an early stage in its development and its subsequent adoption in real-world applications and environments.

We reviewed big data technologies within the context of biomedical research and the adoption of cloud-based architecture by processes geared for big omics data analytics. The advent of cloud technologies capable of handling big data offers the opportunity for efficient, scalable, and secure biomedical data analysis. While reviewing the current omics data processing and analysis landscape, we noted significant challenges related to the need to perform systematic scalable large-scale multi-omics integrative analytics encompassing data handling and storage demands. Currently, the available cloud infrastructures face significant challenges related to providing the necessary resources to handle the rapidly increasing, heterogeneous, and large-scale omics data. These challenges directly affect our ability to harness available resources to understand disease pathobiology and pathophysiology better, ultimately identifying multifactorial genetic disease–related biomarkers for advanced personalized and targeted health care solutions. Undeniably, therefore, there is a need to develop novel standardized approaches that will cater efficient multimodal multi-omics integrative analytics that are exploiting cloud computing infrastructures that are increasingly edging us closer to the tantalizing potential of a sustainable, secure, scalable, and cost-effective technology that can address this challenge.

## Supplemental Material

sj-pdf-1-bbi-10.1177_11779322211035921 – Supplemental material for Cloud Computing Enabled Big Multi-Omics Data Analytics Click here for additional data file. Supplemental material, sj-pdf-1-bbi-10.1177_11779322211035921 for Cloud Computing Enabled Big Multi-Omics Data Analytics by Saraswati Koppad, Annappa B, Georgios V Gkoutos and Animesh Acharjee in Bioinformatics and Biology Insights
