# Supplementary material for: Cloud Computing Enabled Big Multi-Omics Data Analytics
Source: Bioinform Biol Insights. 2021 Jul 28;15:11779322211035921. doi: 10.1177/11779322211035921 (PMC8323418; doi:10.1177/11779322211035921)
Supplement: sj-pdf-1-bbi-10.1177_11779322211035921 – Supplemental material for Cloud Computing Enabled Big Multi-Omics Data Analytics [file sj-pdf-1-bbi-10.1177_11779322211035921.pdf]

Review article

Supplementary Material:
Cloud computing enabled big multi-omics data analytics

Saraswati Koppad[1], Annappa B[1] , Georgios V. Gkoutos[2,3,4,5,6,7] and Animesh Acharjee [2,3,4]*

[1]Department of Computer Science and Engineering, National Institute of Technology Karnataka Surathkal, India -575025 ; saraswatikoppad@gmail.com , annappa@ieee.org

[2]College of Medical and Dental Sciences, Institute of Cancer and Genomic Sciences, Centre for Computational Biology, University of Birmingham, B15 2TT, UK. ; G.Gkoutos@bham.ac.uk, a.acharjee@bham.ac.uk

[3]Institute of Translational Medicine, University Hospitals Birmingham NHS, Foundation Trust, B15 2TT, UK.

[4]NIHR Surgical Reconstruction and Microbiology Research Centre, University Hospital, Birmingham, B15 2WB

[5]MRC Health Data Research UK (HDR UK)

[6]NIHR Experimental Cancer Medicine Centre, B15 2TT, Birmingham, UK

[7]NIHR Biomedical Research Centre, University Hospital Birmingham, Birmingham, B15 2TT, UK

*Correspondence: a.acharjee@bham.ac.uk;

Table S1. Examples of -omics data sources

Source	Description	URL
The Cancer Genome Atlas (TCGA)	Genotype: cancer whole genome and exome sequencing<br>Transcriptome: RNASeq (mRNA, miRNA)<br>Epigenomics: methylation<br>Proteomics: RPPA<br>Phenotype: pathology reports	https://portal.gdc.cancer.gov/
Gene Expression Omnibus (GEO)	Gene expression profiling by microarray or next-generation sequencing<br>Non-coding RNA profiling by microarray or next-generation sequencing<br>Chromatin immunoprecipitation (ChIP) profiling by microarray or next-generation sequencing)<br>Genome methylation profiling by microarray or next-generation sequencing<br>High-throughput RT-PCR<br>Genome variation profiling by array (arrayCGH)<br>SNP arrays<br>Serial Analysis of Gene Expression (SAGE)<br>Protein arrays	https://www.ncbi.nlm.nih.gov/geo/
International Cancer Genome Consortium (ICGC)	Genotype: cancer whole genome and exome sequencing<br>Phenotype: pathology reports	https://icgc.org/
Genomics of Drug Sensitivity in Cancer (GDSC)	Whole genome sequencing<br>Copy number alteration<br>Gene expression<br>Methylation<br>Drug screening data	https://www.cancerrxgene.org
Catalogue of Somatic Mutations in Cancer (COSMIC)	Tumor samples<br>Observed coding mutations<br>Observed gene fusions<br>Copy number variants<br>Gene expression variants<br>Differentially methylated CpGs<br>Non-coding variants<br>Structural mutations<br>Whole genomes	https://cancer.sanger.ac.uk
Cancer Cell Line Encyclopedia (CCLE)	Genotype: whole genome sequencing; whole exome sequenc-	https://portals.broadinstitute.org/ccle

	ing<br>Transcriptome: RNA sequencing	
Genotype-Tissue Expression (GTEx)	Genotype: SNP arrays, whole genome, exome<br>Transcriptome: RNASeq<br>Phenotype: compressive profiles of subjects	https://gtexportal.org
